# Predictive Modeling of Lapses in Care for People Living with HIV in Chicago: Algorithm Development and Interpretation

**DOI:** 10.2196/43017

**Published:** 2023-05-17

**Authors:** Joseph A Mason, Eleanor E Friedman, Samantha A Devlin, John A Schneider, Jessica P Ridgway

**Affiliations:** 1 The Chicago Center for HIV Elimination Department of Medicine University of Chicago Chicago, IL United States

**Keywords:** HIV, predictive model, lapse in care, retention in care, people living with HIV, Chicago, HIV care continuum, electronic health record, EHR

## Abstract

**Background:**

Reducing care lapses for people living with HIV is critical to ending the HIV epidemic and beneficial for their health. Predictive modeling can identify clinical factors associated with HIV care lapses. Previous studies have identified these factors within a single clinic or using a national network of clinics, but public health strategies to improve retention in care in the United States often occur within a regional jurisdiction (eg, a city or county).

**Objective:**

We sought to build predictive models of HIV care lapses using a large, multisite, noncurated database of electronic health records (EHRs) in Chicago, Illinois.

**Methods:**

We used 2011-2019 data from the Chicago Area Patient-Centered Outcomes Research Network (CAPriCORN), a database including multiple health systems, covering the majority of 23,580 people with an HIV diagnosis living in Chicago. CAPriCORN uses a hash-based data deduplication method to follow people across multiple Chicago health care systems with different EHRs, providing a unique citywide view of retention in HIV care. From the database, we used diagnosis codes, medications, laboratory tests, demographics, and encounter information to build predictive models. Our primary outcome was lapses in HIV care, defined as having more than 12 months between subsequent HIV care encounters. We built logistic regression, random forest, elastic net logistic regression, and XGBoost models using all variables and compared their performance to a baseline logistic regression model containing only demographics and retention history.

**Results:**

We included people living with HIV with at least 2 HIV care encounters in the database, yielding 16,930 people living with HIV with 191,492 encounters. All models outperformed the baseline logistic regression model, with the most improvement from the XGBoost model (area under the receiver operating characteristic curve 0.776, 95% CI 0.768-0.784 vs 0.674, 95% CI 0.664-0.683; *P*<.001). Top predictors included the history of care lapses, being seen by an infectious disease provider (vs a primary care provider), site of care, Hispanic ethnicity, and previous HIV laboratory testing. The random forest model (area under the receiver operating characteristic curve 0.751, 95% CI 0.742-0.759) revealed age, insurance type, and chronic comorbidities (eg, hypertension), as important variables in predicting a care lapse.

**Conclusions:**

We used a real-world approach to leverage the full scope of data available in modern EHRs to predict HIV care lapses. Our findings reinforce previously known factors, such as the history of prior care lapses, while also showing the importance of laboratory testing, chronic comorbidities, sociodemographic characteristics, and clinic-specific factors for predicting care lapses for people living with HIV in Chicago. We provide a framework for others to use data from multiple different health care systems within a single city to examine lapses in care using EHR data, which will aid in jurisdictional efforts to improve retention in HIV care.

## Introduction

### Background

Retention in HIV care is an important step in the HIV care continuum for both public health efforts to end the HIV epidemic and for the individual health of people living with HIV [1]. The Centers for Disease Control and Prevention aims to reduce new HIV infections by at least 90% over the next 10 years [2]. Out of every 10 cases of HIV transmission, 8 are associated with people living with HIV who are not engaged in regular HIV medical care [2]. As part of the goal to reduce new HIV infections, it is important to ensure people living with HIV are receiving regular medical care. Currently, only 50.1% of people living with HIV in the United States are receiving continuous HIV care [3]. Therefore, increasing the percentage of people living with HIV retained in care is critical to ending the HIV epidemic in the United States.

Many methods to improve retention in care are being piloted across the United States [4]. In HIV clinical settings, the use of case managers to assist with appointment scheduling and health care navigation has been shown to improve retention [5]. Another initiative, Data to Care, is a strategy by public health departments to use HIV surveillance data to identify people living with HIV that have fallen out of care and attempt to relink them [6]. However, a study on the Data to Care program in Seattle and King County, Washington, found that only 50% of people living with HIV were successfully reengaged in care, suggesting a need for additional strategies to increase the number of people living with HIV in care [7]. One upstream intervention would be to identify people living with HIV who are more likely to fall out of care prior to lapsing in care, allowing them to be prioritized for intense case management and other retention interventions.

### Prior Work

Previous studies have created predictive models to identify people who are likely to be at risk for lapses in HIV care. Ramachandran et al [8] used electronic health record (EHR) data from a single urban HIV clinic to predict retention in care. Similarly, Ridgway et al [9] used EHR data from multiple HIV clinics across the United States to predict retention in care. These studies identified factors associated with lapses in HIV care at the clinic or national level, but public health strategies to improve retention in care in the United States often occur at the city or county level. Local socio-structural factors may impact retention in care among people living with HIV within different cities in unique ways. To our knowledge, no prior studies have predicted which patients will lapse from HIV care within a single urban city (ie, using EHR data from multiple clinics within a single city or geographic region). Our study focuses on the city of Chicago, which is located in Cook County, Illinois, a priority jurisdiction for the Centers for Disease Control and Prevention’s Ending the HIV Epidemic initiative [10]. The Chicago Department of Public Health (CDPH) is responsible for identifying and implementing HIV public health strategies, including the Ending the HIV Epidemic initiative, for both HIV prevention and HIV treatment within the city.

### Goal of This Study

This study aimed to develop predictive models for determining which patients are at risk for lapses in HIV care in Chicago using the Chicago Area Patient-Centered Outcomes Research Network (CAPriCORN). CAPriCORN contains EHR data from a diverse group of 11 different health care systems in Chicago, including academic medical centers, community hospitals, and community health clinic networks [11]. Importantly, CAPriCORN contains data for 12.8 million people, including the majority of 23,580 diagnosed people living with HIV in the city of Chicago [12,13]. Data from CAPriCORN allowed us to follow people living with HIV who obtain HIV care across the different CAPriCORN health care facilities in Chicago. We used these data to identify those engaged in HIV care and to build predictive models to identify people living with HIV at risk for lapses in HIV care within the city of Chicago.

## Methods

### Study Population

We used deidentified EHR data obtained from 7 sites in CAPriCORN, selecting records from adults (aged 18 years and older) with encounters between January 1, 2011, and September 5, 2019. The hospitals included in the CAPriCORN research database use several different EHR systems, including Epic, Cerner, GE Centricity, eClinicalWorks, Sunquest, and Veterans Health Information Systems and Technology Architecture [11]. CAPriCORN was responsible for all harmonization of data across different EHR systems. Data from CAPriCORN are linked by using a hash-based data deduplication method, allowing researchers to identify and follow patients across the different Chicago health care systems [11]. All EHR data were provided at the encounter level, allowing us to incorporate changes to sociodemographic, medical diagnoses, and laboratory information over time. Data contained sociodemographic information, encounter information, diagnosis codes, laboratory data, and antiretroviral therapy (ART) medication or prescription drug orders. We used the following criteria to determine if an individual was HIV-positive: (1) a positive HIV laboratory test (confirmatory HIV antibody, p24 antigen, or HIV viral load > 20 copies/mL), (2) an HIV viral load test performed (regardless of results) and prescription for ART, excluding pre-exposure prophylaxis prescriptions, (3) an HIV diagnosis code (International Classification of Diseases [ICD] version 9 [ICD-9] codes 42, 079.53, 795.71, and V08; ICD version 10 [ICD-10] codes B20, R75, and Z21) and a prescription for ART, excluding pre-exposure prophylaxis, or (4) an HIV diagnosis code and 2 HIV viral load tests performed [14]. Individuals were only included in the study if they met one (or more) of the above criteria and had at least two HIV care encounters. An HIV care encounter was defined as an ambulatory encounter that was either (1) with an infectious disease (ID) provider (taxonomy code “207RI0200X” or “2080P0208X”), or (2) associated with an HIV viral load test or CD4 T cell test. This definition allowed for the inclusion of both people living with HIV who receive their HIV care from their primary care physician as well as those who receive HIV care from their ID physician. To avoid double counting of care visits, we considered visits and laboratory tests within 7 days of each other to be part of the same encounter.

### Ethics Approval

This study was approved by the Chicago Area Institutional Review Board (IRB #00009693).

### Outcome

The outcome of interest in all models was a lapse in HIV care, defined as not having a second HIV care encounter within 1 year after the original HIV care encounter. Because people living with HIV with stable viral loads may have longer time periods between their appointments (eg, from 6 to 9 months), a 1-year gap was chosen to distinguish between these patients and people living with HIV who had truly lapsed in care [9,15,16]. People living with HIV were censored from the study when <12 months of study time remained to ensure that all participants had the opportunity to be identified as having a lapse in care.

### Predictor Variables

We initially created a list of 134 variables for inclusion in the models; however, 20 variables were excluded in the final models due to multicollinearity between these variables and those already in the model. Final models were built using 114 variables including patient demographics, history of lapse in care, site, provider specialty, insurance information, laboratory tests and results, ART medications, and patient diagnoses. Medical diagnoses were based on the presence of the ICD-9 and ICD-10 diagnosis codes. Specifically, we examined diagnoses of cancer, cardiovascular disease, diabetes, mental health disorders (including mood and anxiety disorders), obesity, sexually transmitted infections (STIs; ie, chlamydia, gonorrhea, hepatitis B, hepatitis C, and syphilis), as well as the use of alcohol, cannabis, opioids, sedatives, stimulants, and tobacco. CAPriCORN provided racial demographic information, with patients able to select more than one race. Laboratory test variables included HIV viral load, CD4 count, HIV genotype, STI tests, and other ID tests within the preceding 12 months. The complete list of variables, including those excluded due to multicollinearity, as well as their ICD-9 or ICD-10 codes, is available in Multimedia Appendices 1 and 2.

### Model Development

Encounter data were randomly split into a training set (70% of total data) and a validation set (30% of total data). The split was performed while holding a constant ratio of the outcome variable. We created a baseline logistic regression model using only patient demographics and previous history of lapses in care allowing us to quantify how much more predictive capability was provided by including additional data related to laboratory tests and diagnoses. We then created 4 different predictive models: logistic regression, elastic net logistic regression, random forest, and XGBoost. The hyperparameters for the elastic net, random forest, and XGBoost models were tuned using a grid search and can be found in Multimedia Appendix 3. The hyperparameters were optimized using 5-fold cross-validation on the metric of the area under the receiver operating characteristic curve (AUC).

### Model Performance

The performance of these 4 models was compared with each other and with the baseline logistic regression model. Model performance was evaluated using AUC via DeLong’s [17] method. Variable influence for each model was evaluated using the absolute value of the *t* statistic for the baseline and full logistic regression models, the value of coefficients for the elastic net logistic regression, the Gini index for the random forest, and the relative influence for the XGBoost model [18,19]. Each model can provide the association of variables; however, unlike the baseline regression, full logistic regression, and elastic net logistic regression models, the random forest and XGBoost models we created cannot provide the directionality of association. All analyses were performed using R (version 4.0.3; R Core Team).

## Results

### Study Population

The study population contained 16,930 people living with HIV with a total of 191,492 HIV care encounters during the study period. When examining the demographic information for participants based on information provided at their last HIV care encounter, people living with HIV in this study were predominantly male (75.2%, 12,733/16,930), Black (61.4%, 10,395/16,930), non-Hispanic or -Latino (84.2%, 14,249/16,930), and had all HIV care encounters at a single site within CAPriCORN (95.3%, 16,135/16,930). Based on each participant’s last 2 HIV care encounters, 25.1% (4257/16,930) of participants had a lapse in care (Table 1). Over the study period, 42.1% (7135/16,930) of participants had a change in their lapse in HIV care status (either from engaged to lapsed in care or vice versa). The different care sites served a wide range of number of patients; the smallest site served 224 unique people living with HIV, and the largest site served 10,035 unique people living with HIV. The proportion of lapses in care varied among sites. The worst-performing site was Site F, with 21.4% (61/292) of encounters resulting in a lapse in care. The best-performing site was Site G, with 3.7% (5283/141,864) of encounters resulting in a lapse in care. Over the study period, 94.4% (180,777/191,492) of HIV care encounters were followed by a second HIV care encounter within 1 year.

**Table 1:** Characteristics of people living with HIV in the Chicago Area Patient-Centered Outcomes Research Network in Chicago, Illinois, 2011-2019 (N=16,930).

Variable of interest and values		Frequency, n (%)
**Lapses in HIV care**		
	No lapse in care	12,673 (74.9)
	Lapse in care	4257 (25.1)
**Sex**		
	Male	12,733 (75.2)
	Female	4147 (24.5)
	Unknown	50 (0.3)
**Race**		
	African American or Black	10,395 (61.4)
	White	5168 (30.5)
	Multiracial	136 (0.8)
	Other	276 (1.6)
	Unknown	955 (5.6)
**Ethnicity**		
	Hispanic or Latino	2681 (15.8)
	Non-Hispanic or -Latino	14,249 (84.2)
**Age (years)**		
	Less than 25	868 (5.1)
	25 to less than 35	3080 (18.2)
	35 to less than 45	3520 (20.8)
	45 to less than 55	4587 (27.1)
	55 or older	4875 (28.8)
**Primary payer type**		
	Private	3030 (17.9)
	Medicare	2861 (16.9)
	Medicaid	5162 (30.5)
	Other	1031 (6.1)
	Unknown	4846 (28.6)
**Site**		
	Multisite	795 (4.7)
	Single site	16,135 (95.3)
**Comorbidities**		
	Hypertension	5128 (30.3)
	Mental health	5757 (34.0)
	Tobacco	4068 (24.0)
	Mood disorder	3914 (23.1)
	Alcohol	1347 (8.0)
	Opioids	1114 (6.6)
**Gonorrhea laboratory test in the past year**		
	Yes	2157 (12.7)
	No	14,773 (87.3)
**Chlamydia laboratory test in the past year**		
	Yes	2419 (14.3)
	No	14,511 (85.7)

### Model Performance

**Figure 1:**
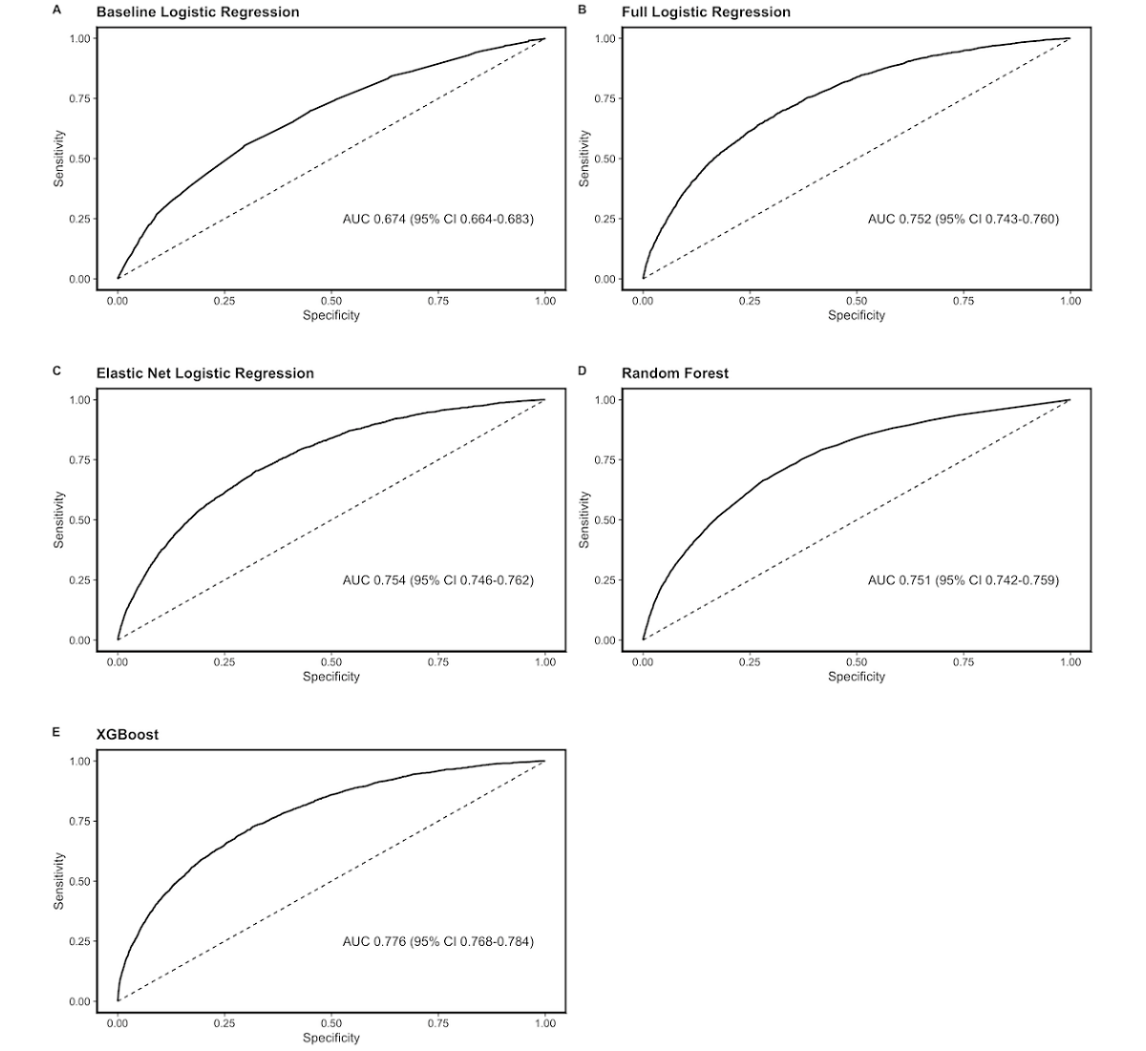
Prediction model receiver operator curves of lapses in care for people living with HIV in Chicago, Illinois, 2011-2019. AUC: area under the receiver operating characteristic curve.

Using our validation data set containing 57,447 encounters, all models created using 114 variables outperformed the baseline logistic regression model. The greatest improvement in AUC was seen with the XGBoost model (AUC 0.776, 95% CI 0.768-0.784 vs 0.674, 95% CI 0.664-0.683 for the baseline model; *P<*.001; Figure 1).

The XGBoost model had a significant difference in performance between the full logistic regression (AUC 0.776, 95% CI 0.768-0.784 vs 0.752, 95% CI 0.743-0.760; *P*<.001), elastic net logistic regression (AUC 0.776, 95% CI 0.768-0.784 vs 0.754, 95% CI 0.746-0.762; *P*<.001), and random forest (AUC 0.776, 95% CI 0.768-0.784 vs 0.751, 95% CI 0.742-0.759; *P*<.001) models. There was a significant difference in performance between the elastic net logistic regression model and the full logistic regression model (AUC 0.754, 95% CI 0.746-0.762 vs 0.752, 95% CI 0.743-0.760; *P*<.001); however, the magnitude of the difference between the 2 models was slight and may not be clinically meaningful. There was no significant difference in performance between the elastic net logistic regression model and the random forest model (AUC 0.754, 95% CI 0.746-0.762 vs 0.751, 95% CI 0.742-0.759; *P*=.43; Multimedia Appendix 4).

### Predictor Variable Importance

The top 10 most influential predictors in each model are listed in Tables 2 and 3. Although each model had slightly different results, common predictors included no lapse in care in the prior year, being seen by an ID provider (vs a primary care provider), and Hispanic or Latino ethnicity. Influential predictors for the full logistic regression model were no lapse in care in the prior year (adjusted odds ratio [aOR] 0.42, 95% CI 0.39-0.46; *P*<.001), being seen by an ID provider (vs a primary care provider; aOR 0.54, 95% CI 0.49-0.58; *P*<.001), age of 55 years or older (vs 25 years to less than 35 years; aOR 0.56, 95% CI 0.51-0.61; *P*<.001), Hispanic ethnicity (aOR 0.57, 95% CI 0.52-0.63; *P*<.001), age of 45 years to <55 years (vs 25 years to less than 35 years; aOR 0.68, 95% CI 0.63-0.73; *P*<.001), and site F (aOR 6.39, 95% CI 3.89-10.47; *P*<.001).

**Table 2:** Most influential variables for baseline logistic regression and full logistic regression models of lapses in care for people living with HIV in Chicago, Illinois, 2011-2019.

Model and variable		aOR^a^ (95% CI)	*P* value
**Baseline** **logistic regression**			
	No lapse in care in the prior 12 months	0.34 (0.32-0.36)	<.001
	Hispanic ethnicity	0.32 (0.29-0.34)	<.001
	Age of 55 years or older	0.53 (0.49-0.57)	<.001
	Black race	0.41 (0.37-0.46)	<.001
	Age of 45 years to less than 55 years	0.68 (0.63-0.73)	<.001
	Unknown race	1.72 (1.52-1.94)	<.001
	White race	0.63 (0.56-0.70)	<.001
	Asian race	0.40 (0.31-0.51)	<.001
	Age of 35 years to less than 45 years	0.86 (0.80-0.93)	<.001
	Multiracial race	0.64 (0.41-0.99)	.047
**Full logistic regression**			
	No lapse in care in the prior 12 months	0.42 (0.39-0.46)	<.001
	Infectious disease provider	0.54 (0.49-0.58)	<.001
	Age of 55 years or older	0.56 (0.51-0.61)	<.001
	Hispanic ethnicity	0.57 (0.52-0.63)	<.001
	Age of 45 years to less than 55 years	0.68 (0.63-0.73)	<.001
	Site F	6.39 (3.89-10.47)	<.001
	CD4 T cell percentage result greater than or equal to 28 in the prior 12 months	1.36 (1.25-1.48)	<.001
	Gonorrhea laboratory test in the prior 12 months	0.35 (0.26-0.47)	<.001
	Primary insurance unknown	0.56 (0.47-0.66)	<.001
	Cancer diagnosis ever	0.78 (0.72-0.83)	<.001

^a^aOR: adjusted odds ratio.

**Table 3:** Top performing variables for elastic net logistic regression, random forest, and XGBoost models of lapses in care for people living with HIV in Chicago, Illinois, 2011-2019.

Model and variable		Variable importance score
**Elastic net logistic regression**		
	Site F	1.575
	No lapse in care in the prior 12 months	–0.835
	Chlamydia laboratory test in the prior 12 months	0.815
	Site A	0.814
	Gonorrhea laboratory test in the prior 12 months	–0.769
	Site C	0.649
	Infectious disease provider	–0.583
	Hispanic ethnicity	–0.512
	Primary insurance unknown	–0.506
	Emergency medicine provider	0.504
**Random forest**		
	Primary insurance Medicaid	216.8
	No lapse in care in the prior 12 months	205.9
	Hypertension diagnosis ever	200.0
	Age 35 years to less than 45 years	195.0
	Tobacco diagnosis ever	194.5
	Age of 45 years to less than 55 years	193.0
	Mental health diagnosis ever	190.5
	CD4 T cell percentage result less than 28 in the prior 12 months	184.1
	Mood disorder diagnosis ever	172.5
	Syphilis diagnosis ever	171.4
**XGBoost**		
	Site G	0.089
	No lapse in care in the prior 12 months	0.065
	Infectious disease provider	0.045
	Site A	0.038
	Previous viral load result suppressed	0.031
	Age of 55 years or older	0.019
	Hispanic ethnicity	0.019
	Primary insurance Medicaid	0.019
	CD4 T cell percentage result greater than or equal to 28 in the prior 12 months	0.018
	HIV viral load laboratory result unsuppressed in the last 12 months	0.017

## Discussion

### Principal Results

Our study found that 25.1% (4257/16,930) of patients experienced a lapse in care based on their last eligible HIV care encounter, which is lower than the CDPH estimate of 33.8% of people living with HIV in Chicago not accessing HIV care [13]. Our lower percentage is likely due to two factors: (1) a slightly different patient care population and (2) different definitions being used to determine who was not in care. We defined a lapse in HIV care as not having a second HIV care encounter within 1 year after the original HIV care encounter; however, CDPH defines a lapse in HIV care as not having at least one HIV laboratory test (CD4 or viral load) within a year [13]. Additionally, we only calculated lapses in care among patients with at least two HIV care encounters, rather than among all people living with HIV within the jurisdiction of Chicago identified in the enhanced HIV/AIDS Reporting System used by CDPH [13]. By limiting our study population to people with at least two HIV care encounters, we may have selected patients with a history of retention in care, resulting in a patient population less likely to experience a lapse in care.

In this study, we developed predictive models for lapses in care among people living with HIV using linked data that incorporated information from multiple health care systems across Chicago. Our XGBoost model outperformed our baseline model, which included only information about demographics and past lapses in care. Across the different models, we assessed various HIV health care and STI-related factors in predicting the likelihood of lapses in HIV care. In all models, prior lapses in care were among the most influential predictors of future lapses in care. This observation aligns with other studies that have found previous retention in care to be important in predicting future retention [8,9,20]. Our models also showed that higher CD4 T cell counts are associated with an increased risk of lapsing in care, supporting previous findings [21-23]. A previous suppressed HIV viral load laboratory result and an unsuppressed HIV viral load laboratory result in the prior 12 months were found to be associated with lapsing in care; however, the directionality could not be determined from the XGBoost model. A gonorrhea laboratory test in the last year was predictive of a decreased risk of lapsing in care in the logistic regression model and the elastic net logistic regression model. A chlamydia laboratory test in the last year was predictive of an increased risk of lapsing in care in the elastic net logistic regression model.

In addition to HIV-related factors, we also examined the ability of sociodemographic characteristics to predict lapses in HIV care. Since CAPriCORN covers the majority of people living with HIV in Chicago, this is a unique opportunity to view how these factors impact lapses in care in Chicago. We found that younger age is a predictor for lapses in care, which has been seen in other studies [9,21,22,24-26]. Hispanic or Latino ethnicity was associated with a decreased risk of lapsing in care in this study, a finding supported by some previous literature but contradicted by others [9,21,22,25-28]. Another predictor for not lapsing in care was the female sex, consistent with previous studies [22,28].

In terms of general medical and health care factors, we found that people living with HIV with chronic comorbidities, particularly cancer, are less likely to lapse in care, as others have also found [23]. This lower risk of lapsing in care may result from people with other comorbidities being more likely to follow up with both their chronic disease health care provider as well as their ID provider [9,29]. The comorbidity of hypertension (having hypertension or being hypertension-free) was also associated with lapsing in care [9,21]. Unfortunately, this result comes from the random forest model, so we cannot assess the directionality of the association. Finally, despite examining data from a single city, we found that the HIV care site was associated with a higher risk of lapsing in care. The HIV care sites included in our study ranged from a small percentage to almost a fifth of encounters resulting in a lapse of care, which could be due to a myriad of clinic-level factors, such as different patient demographics, acceptance of multiple insurance types, provider familiarity and trust, different neighborhood locations, and varying levels of resources available for identifying patients at risk for lapses in care and reengaging them. Additionally, structural racism and disinvestment in certain neighborhoods in Chicago have disproportionately impacted marginalized communities and the clinics that serve these communities, which may also be reflected in clinic site differences [30]. Our findings regarding the impact of chronic comorbidity and clinic-level factors on risk for lapsing in care suggest that public health departments may benefit from using data from city-wide research networks like CAPriCORN to better understand which people living with HIV have access to HIV care and are engaged in care.

Consequently, our model could allow public health agencies to identify which groups of people living with HIV within Chicago are at the highest risk of lapsing in care, allowing resources to be targeted toward these patients *before* they lapse in care. The integration of research data with city- or county-based HIV surveillance data can provide a more complete picture of access to and engagement with HIV care than either data source alone. For instance, CDPH, like most city public health departments, can follow the entire population of people living with HIV in Chicago due to mandatory laboratory reporting of HIV tests. CDPH also has precise knowledge of who enters or leaves the jurisdiction. This information was unavailable to us in the CAPriCORN health network. However, CAPriCORN contains a wide range of information derived from the EHRs of patients, encompassing their entire range of medical illnesses and test results, instead of the public health departments’ relatively limited HIV-related medical information. City health departments typically use viral load and CD4 count laboratory tests as proxies for HIV care visits, whereas we gathered information from both laboratory and clinic visits, allowing us to capture the full range of HIV care encounters and providing a unique way to look at the city-wide continuum of care. For instance, using the CAPriCORN data, we were able to determine that most patients receive care from a single site; only 4.7% (795/16,930) of patients received care from more than one site within the research network. This is less than a study that found that 8% of people living with HIV in Philadelphia received HIV care from multiple sites [31]. Having the ability to determine this important information about the care continuum demonstrates the benefits of incorporating data from EHRs through research networks like CAPriCORN into public health surveillance for HIV care.

### Limitations

There are several limitations to this study. In order to deidentify the data set and protect patient privacy, CAPriCORN removed specific dates of encounters. Therefore, we used patient age in days to determine the relative time between encounters. The lack of dates also means that we cannot account for study-wide temporal trends. Some of our sites also had incomplete data. For example, 1 site was missing HIV laboratory data, resulting in no eligible HIV care encounters, meaning that we were unable to incorporate this site into our models. This incompleteness of data shows a larger limitation of city-wide research networks. It is critical for cities using our framework to focus on incorporating high-quality data, ensuring the most accurate and complete data are available for tracking engagement in HIV care. Additionally, we determined that an HIV care encounter included primary care visits in which an HIV viral load test or CD4 test were performed. However, it is possible that some primary care encounters were for HIV care but did not include performing an HIV viral load test or CD4 test leading to misclassifications. Similarly, we focused on 1 definition of a lapse in HIV care as our outcome, but there are multiple standards for defining a lapse in HIV care [32]. Future studies should compare different standards, such as the Health Resources and Services Administration HIV/AIDS Bureau measure [33]. As we did not have access to individual patient records for review of medical records, we used ICD-9/10 codes to identify diagnoses, which might lack sensitivity and specificity in identifying patients with medical comorbidities. However, ICD-9/10 codes provide a ubiquitous way for researchers to build predictive models using easily accessible data even if they lack the resources to carry out a review of medical records for every patient. Previous studies have shown that using ICD-9/10 codes can be effective at scaling up the rapid identification of people living with HIV [14,34]. Future studies should include social history variables and unstructured fields or free-text notes to see if they improve overall model performance. This model is possible because of the existence of the CAPriCORN research network. For this work to be extended in other settings, a deidentified, centralized data source from multiple hospital systems would need to be created in other jurisdictions. Our study is retrospective, so the performance of the model in a prospective setting should be tested in future studies prior to implementation.

### Conclusions

We built predictive models for lapses in HIV care for people living with HIV from 7 different health care systems across Chicago and identified health care factors found in structured EHR data that are important for predicting lapses in care. Our models also serve as a proof of concept to show that differences in site-based practices (ie, laboratory tests ordered, diagnosis codes, different EHR systems, etc) do not prevent the ability to develop predictive models for lapses in care within a city. To our knowledge, this is the first time predictive models of lapses in HIV care have been built using EHR data from a large research network of health care systems within a major urban city with a high HIV prevalence rate. By focusing on data from patients served by a single public health department jurisdiction, findings from this study may inform HIV public health strategies for the city of Chicago. This model framework can also be adapted and used in clinics to identify patients most at risk for lapses in care who would benefit from tailored interventions or resources, thereby mitigating the number of people living with HIV who fall out of care and supporting efforts to end the HIV epidemic.

## Appendix

Multimedia Appendix 1 Clinical variables included in prediction models of lapses in care for people living with HIV in Chicago, Illinois, 2011-2019.

Multimedia Appendix 2 ART codes included in prediction models of lapses in care for people living with HIV in Chicago, Illinois, 2011-2019.

Multimedia Appendix 3 Hyperparameter values used in prediction models of lapses in care for people living with HIV in Chicago, Illinois, 2011-2019.

Multimedia Appendix 4 Sensitivity and specificity values at different cut-off points for prediction models of lapses in care for people living with HIV in Chicago, Illinois, 2011-2019.

## Abbreviations

aOR: adjusted odds ratio
ART: antiretroviral therapy
AUC: area under the receiver operating characteristic curve
CAPriCORN: Chicago Area Patient-Centered Outcomes Research Network
CDPH: Chicago Department of Public Health
EHR: electronic health record
ICD: International Classification of Diseases
ID: infectious disease
STI: sexually transmitted infection
