# Supplementary material for: Predictive Modeling of Lapses in Care for People Living with HIV in Chicago: Algorithm Development and Interpretation
Source: JMIR Public Health Surveill. 2023 May 17;9:e43017. doi: 10.2196/43017 (PMC10233431; doi:10.2196/43017)
Supplement: Multimedia Appendix 3 [file publichealth_v9i1e43017_app3.docx]

| Model | Hyperparameter | Value |
| --- | --- | --- |
|  |  |  |
| Elastic Net Logistic Regression | Ratio between L_1_ and L_2_ penalty | 0.55 |
| Random Forest | Number of variables to randomly sample as candidates at each split | 14 |
| XGBoost | Maximum number of iterations | 1,000 |
| XGBoost | Maximum depth of a tree | 10 |
| XGBoost | Step size shrinkage used in update to prevent overfitting | 0.01 |
| XGBoost | Minimum loss reduction required to make a split | 5 |
| XGBoost | Subsample ratio of columns when constructing each tree | 0.7 |
| XGBoost | Subsample ratio of training instances | 0.7 |
| XGBoost | Minimum sum of weights of all observations required in a child | 1 |
