# Supplementary material for: Predictive Modeling of Lapses in Care for People Living with HIV in Chicago: Algorithm Development and Interpretation
Source: JMIR Public Health Surveill. 2023 May 17;9:e43017. doi: 10.2196/43017 (PMC10233431; doi:10.2196/43017)
Supplement: Multimedia Appendix 4 [file publichealth_v9i1e43017_app4.docx]

| Model | Model Cutoff | Sensitivity  (%, 95% CI) | Specificity  (%, 95% CI) |
| --- | --- | --- | --- |
|  |  |  |  |
| Baseline Logistic Regression |  |  |  |
|  | ≥ 10 | 23 (21, 24) | 93 (92, 93) |
|  | ≥ 09 | 29 (27, 30) | 90 (89, 90) |
|  | ≥ 08 | 33 (31, 34) | 87 (87, 87) |
|  | ≥ 07 | 34 (32, 35) | 86 (86, 87) |
|  | ≥ 06 | 55 (54, 57) | 70 (70, 71) |
|  | ≥ 05 | 70 (68, 71) | 55 (55, 56) |
|  | ≥ 04 | 84 (83, 85) | 36 (36, 37) |
|  | ≥ 03 | 95 (94, 96) | 15 (14, 15) |
| Logistic Regression |  |  |  |
|  | ≥ 10 | 42 (40, 43) | 88 (87, 88) |
|  | ≥ 09 | 46 (45, 48) | 86 (85, 86) |
|  | ≥ 08 | 51 (49, 53) | 83 (82, 83) |
|  | ≥ 07 | 56 (54, 58) | 79 (79, 79) |
|  | ≥ 06 | 63 (61, 64) | 74 (74, 74) |
|  | ≥ 05 | 70 (69, 72) | 66 (66, 67) |
|  | ≥ 04 | 79 (77, 80) | 57 (56, 57) |
|  | ≥ 03 | 89 (87, 90) | 42 (41, 42) |
| Elastic Net Logistic Regression |  |  |  |
|  | ≥ 10 | 41 (40, 43) | 88 (88, 88) |
|  | ≥ 09 | 46 (44, 48) | 86 (85, 86) |
|  | ≥ 08 | 51 (49, 53) | 83 (82, 83) |
|  | ≥ 07 | 57 (55, 58) | 79 (79, 79) |
|  | ≥ 06 | 63 (61, 65) | 74 (73, 74) |
|  | ≥ 05 | 71 (70, 73) | 66 (66, 66) |
|  | ≥ 04 | 80 (79, 82) | 55 (55, 56) |
|  | ≥ 03 | 90 (89, 91) | 39 (39, 40) |
| Random Forest |  |  |  |
|  | ≥ 10 | 13 (12, 13) | 58 (56, 59) |
|  | ≥ 09 | 14 (14, 15) | 54 (52, 56) |
|  | ≥ 08 | 17 (16, 17) | 50 (49, 52) |
|  | ≥ 07 | 19 (19, 19) | 47 (45, 48) |
|  | ≥ 06 | 22 (22, 22) | 43 (41, 44) |
|  | ≥ 05 | 26 (25, 26) | 37 (35, 39) |
|  | ≥ 04 | 30 (30, 30) | 32 (30, 34) |
|  | ≥ 03 | 36 (35, 36) | 26 (25, 28) |
| XGBoost |  |  |  |
|  | ≥ 10 | 12 (12, 12) | 54 (52, 56) |
|  | ≥ 09 | 14 (14, 14) | 50 (48, 51) |
|  | ≥ 08 | 17 (17, 17) | 45 (43, 47) |
|  | ≥ 07 | 21 (21, 21) | 40 (38, 41) |
|  | ≥ 06 | 26 (26, 26) | 34 (32, 35) |
|  | ≥ 05 | 33 (33, 33) | 26 (25, 28) |
|  | ≥ 04 | 42 (42, 43) | 19 (18, 21) |
|  | ≥ 03 | 55 (54, 55) | 12 (11, 13) |
